# A Contribution for the Valorisation of Sheep and Goat Cheese Whey through Nanofiltration

**DOI:** 10.3390/membranes8040114

**Published:** 2018-11-20

**Authors:** Antónia Macedo, Joana Monteiro, Elizabeth Duarte

**Affiliations:** 1 Escola Superior Agrária, Polytechnic Institute of Beja, Rua Pedro Soares, Ap. 6158, 7801-908 Beja, Portugal; 2 LEAF—Linking Landscape, Environment, Agriculture and Food, Instituto Superior de Agronomia, University of Lisbon, Tapada da Ajuda, 1349-017 Lisboa, Portugal; eduarte@isa.utl.pt; 3 Instituto Superior de Agronomia, University of Lisbon, Tapada da Ajuda, 1349-017 Lisboa, Portugal; joanna-monteiro@hotmail.com

**Keywords:** sheep cheese whey, goat cheese whey, ultrafiltration permeates, nanofiltration performance

## Abstract

The amount of cheese whey generated from the production of speciality sheep and goat cheese is significantly growing due to the acclaimed nutritional and medicinal benefits of the milk from these species. However, most of the cheese whey generated has no applications, thus giving rise to environmental problems. This work focuses on the study of the performance of the nanofiltration process for recovering the permeates of ultrafiltration from sheep and goat cheese whey. Nanofiltration experiments were carried out with membranes of nanofiltration (NF) in total recirculation and concentration modes, at 25 °C. Nanofiltration of the ultrafiltration permeates from sheep cheese whey was done at a pressure of 3.0 × 10^6^ Pa and a circulation velocity of 1.42 m·s^−1^, until a volume concentration factor (VCF) of 2.5. Nanofiltration of the permeates from ultrafiltration of goat cheese whey was performed at a pressure of 2.0 × 10^6^ Pa and a circulation velocity of 0.94 m·s^−1^, until a VCF of 2.0. From the results, it was concluded that osmotic pressure was the most important factor affecting the performance of the process. In both cases, the final permeates had a much lower organic load and its future use in the process of cheese making should be evaluated.

## 1. Introduction

Cheese whey is an agro-industrial byproduct of the dairy industry, which corresponds to the aqueous remnants from the processing of milk into cheese or casein manufacturing by rennet-driven coagulation [1]. It contains about 55% of the nutrients found in milk, including lactose, soluble proteins, peptides, lipids, minerals, and vitamins [2]. Worldwide cheese whey production is estimated at 180 to 190 million tons per year, although only 50% of it is industrially processed [1,3] by ultrafiltration, followed by diafiltration, into value-added products, such as whey protein concentrates (WPC) and whey protein isolates (WPI) [4]. Moreover, small and traditional sheep and goat cheese making industries that do not process whey further have to manage it as a waste. Moreover, growing interest today in the consumption of milk and milk products from these species, mainly attributed to their lower allergenic and higher nutritional properties (compared to cow milk), has led to an increase in the production of milk from these species. Nowadays, the annual production of sheep and goat milk is 10.2 Mt and 17.8 Mt, corresponding to 1.3% and 2.3% of the worldwide production of milk; however, an increase of 2.5 Mt and 4.1 Mt by the year 2025 is expected from sheep and goat milk, respectively [5]. Since sheep and goat milk are mainly used for the production of speciality cheeses, which are valued for their specific texture and flavour, an increase in their demand may lead to increased world production of cheese whey from these small ruminants. Compared to bovine or sheep, goat cheese whey is richer in oligosaccharides, where the structures are similar to those of human milk and contain sialic acid, which appears to promote the development of an infant’s brain [6]. Furthermore, it is also richer in nonprotein nitrogen compounds, namely nucleotides and free amino acids, making it suitable for the production of baby food, removing the need to add these nutritional components [7]. Sheep milk contains a higher concentration of whey proteins than cow and goat milk [8], and therefore the resulting cheese whey is used, in some dairies, for the production of whey cheeses, through a process of thermal precipitation of the whey proteins. However, the production process of these whey cheeses has low efficiency, since it leads to the release of another byproduct (second cheese whey), which still contains about 50% of the initial protein and almost all the lactose [9,10]. Thus, this is not the best solution for the recovery of valuable components or for the minimisation of the environmental problem. Moreover, due to the thermal energy consumed in this process, this becomes costly, affecting the price of the produced whey cheeses.

To reduce the environmental impact of cheese whey while adding value to an undervalorised dairy sidestream, membrane technology offers a promising and cost-effective strategy. Nanofiltration (NF), due to its specific characteristics, including high selectivity towards small solutes and low energy consumption, can provide a series of advantages over other separation processes, such as reverse osmosis, for recovering valuable solutes from cheese whey, and citing the cost benefit of the lower pressure operations. In dairies, NF has mostly been used for the demineralisation of salted and acid whey or to produce whey demineralised lactose concentrates for the food industry. However, the performance of NF is affected by concentration polarisation due to an accumulation of solutes at the membrane surface and the increase of osmotic pressure. This is mainly caused by lactose and salts concentration, reducing the effective transmembrane pressure. The resulting boundary layer can give rise to irreversible adsorption or precipitation of salts, namely calcium phosphates, at the membrane surface, and this can be a major drawback of this process [11,12]. In milk or ultrafiltration permeates, the concentration of calcium and phosphate ions is very close to the solubility constant such that during the process of concentration by NF, precipitation can occur under favourable environmental conditions, such as high temperature and pH, as was observed by some authors [13]. These phenomena lead to the decline of the permeation fluxes and alter the selectivity of the membranes, contributing to a poor performance of this process.

Bearing in mind that: (i) Sheep and goat cheese whey have excellent and specific nutritional and functional characteristics, as described earlier; (ii) most of the whey delivered in small cheese dairies has no applications, thus causing environmental problems; and (iii) both types of cheese whey present different compositions, such as a higher concentration of whey proteins and fat (especially in sheep cheese whey), compared with bovine whey, their separation through membrane technology still constitutes a challenge. Therefore, this work aims to:

(1) Evaluate the performance of nanofiltration (applied to the permeates of ultrafiltration from sheep and goat cheese whey) for recovering lactose and reducing their final organic load;
(2) Apply mass transfer models to the experimental results for identifying the main causes affecting the performance of the process.

The intended results of this work will be an important contribution to improve existing knowledge on the application of membrane technology to goat and sheep cheese whey, adding value to a product that is still treated as a waste and reducing its environmental impact.

## 2. Mass Transfer Models for Nanofiltration of the Permeates of Ultrafiltration

The permeates from ultrafiltration of cheese whey were nanofiltrated to recover lactose and produce a final permeate with a very low organic load. Based on the model of resistances in series and considering the effect of osmotic pressure and neglecting the resistance due to the formation of a gel, the following equation can be written:

(1) $$J=\frac{\Delta P-\Delta \pi }{\mu \left(R_{m}+R_{f}+R_{\mathrm{cp}}\right)},$$

where J is the permeate flux (m·s^−1^); ΔP is the transmembrane pressure (Pa); Δπ is the difference of osmotic pressure between retentate and permeate (Pa); μ is the viscosity of the permeate solution (Pa·s); R_m_ is the membrane resistance (m^−1^); R_f_ is the fouling resistance (m^−1^); and R_cp_ is the resistance due to concentration polarisation phenomena (m^−1^).

The osmotic pressure of cheese whey permeates is mainly due to lactose, with minor contributions of the cations potassium, sodium, and chloride [14]. In this work, since lactose is the main component of the solids, followed by chloride ions, we consider that the osmotic pressure of these permeates is principally a result of these constituents. During the concentration process, lactose is almost totally retained, but chloride ions preferentially permeate the membranes (the apparent rejection coefficients are 0.2). Therefore, the increase in the concentration of lactose near the membrane surface leads to an increase in osmotic pressure in the retentate, while the high permeation of the chloride ions through the membranes leads to a difference of osmotic pressure across the membranes, which is practically constant during the concentration process. Then, we can write:

(2) $$\pi_{\mathrm{retentate}}={\;\pi }_{\mathrm{NaCl}\;\mathrm{ret}}+\pi_{\mathrm{lacm}},$$

where $\pi_{\mathrm{retentate}}$ is the osmotic pressure of the retentate; $\pi_{\mathrm{NaCl}\;\mathrm{ret}}$ is the osmotic pressure due to chloride in the retentate; and $\pi_{\mathrm{lacm}}$ is the osmotic pressure due to lactose in the polarisation layer near the membrane surface, and

(3) $$\pi_{\mathrm{permeate}}=\pi_{\mathrm{NaCl}\;\mathrm{perm}},$$

where $\pi_{\mathrm{permeate}}$ is the osmotic pressure of the permeate and $\pi_{\mathrm{NaCl}\;\mathrm{perm}}$ is the osmotic pressure caused by chloride in the permeate stream.

Therefore:

(4) $$\Delta \pi =\pi_{\mathrm{retentate}}-\pi_{\mathrm{permeate}}=\left(\pi_{\mathrm{NaCl}\;\mathrm{ret}}-\pi_{\mathrm{NaCl}\;\mathrm{perm}}\right)+\pi_{\mathrm{lacm}}=K+\pi_{\mathrm{lacm}},$$

where K is a constant, corresponding to the difference of osmotic pressure across the membranes due to chloride ions.

Using the van’t Hoff equation to express the variation of osmotic pressure with the concentration of lactose, and since its concentration in permeates is negligible, we can write:

(5) $$\pi_{\mathrm{lacm}}=C_{\mathrm{lacm}}\mathrm{RT},$$

where $\pi_{\mathrm{lacm}}$ is the osmotic pressure due to lactose in the retentate (in the polarisation layer near the membrane); $C_{\mathrm{lacm}}$ is the lactose concentration in the polarisation layer (mol·m^−3^); R = 8.314 J·mol^−1^·K^−1^ is the universal constant of ideal gases; and T is the absolute temperature (K).

Substituting Equations (4) and (5) in Equation (1), we can obtain:

(6) $$J=\frac{\Delta P-K-C_{\mathrm{lacm}}\mathrm{RT}}{\mu \left(R_{m}+R_{f}+R_{\mathrm{cp}}\right)}.$$

According to film theory, the ratio between the concentration of a solute totally retained by membranes in the polarisation layer (C_lacm_) and in the bulk retentate (C_lacb_) is called the concentration polarisation modulus, β [15]. Therefore, substituting this result in Equation (6), we can rewrite this equation as:

(7) $$J=\frac{\Delta P-K}{{\mu \;R}_{t}}-\frac{{\beta \;C}_{\mathrm{lacb}\;}R\;T}{{\mu \;R}_{t}},$$

where $R_{t}$ is the total resistance, which is the sum of $R_{m}$, $R_{f}$ and $R_{\mathrm{cp}}$.

As can be seen in Equation (7), for a given pressure, the permeate flux varies linearly with the solute concentration, considering that: (i) The total resistance remains constant; and (ii) the viscosity of the NF permeate is similar to that of pure water, since lactose and other minor organic compounds (that mostly contribute to the viscosity of these permeates) are almost totally retained by NF membranes. Thus, from the linear equation, it is possible to determine the total resistance and the average concentration polarisation modulus through the ordinate at the origin and the slope, respectively.

## 3. Materials and Methods

### 3.1. Sampling and Storage of Samples

In this work, 27 samples of sheep cheese whey, resulting from the manufacture of sheep cured cheese, were collected for 27 months and with a periodicity of 1 sample per month. All the samples were taken from the same cheese dairy, belonging to the Demarcated Region of Serpa cheese.

A set of six samples of goat cheese whey came from a fresh goat cheese-making, located about 5 km from Beja, Portugal. Samples were collected once a week and a volume of 20 L was collected for the experiments and 1.5 L for physicochemical analyses. Samples were collected in 5 L beakers, immediately after cheese making and transported to the laboratory. The samples were subjected to the pretreatment described in Section 3.2. The ultrafiltration permeates were preserved by the addition of sodium azide, to prevent their biological contamination, and then frozen in a freezing chamber at −27 °C for a period of three days.

### 3.2. Pretreatment of the Samples

#### 3.2.1. Samples of Sheep Cheese Whey

Soon after its reception, sheep cheese whey was filtered twice through cotton cloths (used in traditional small dairies) to remove suspended solids and casein fines. After, it was heated to a temperature of 35 °C and then skimmed into a Westfalia brand centrifuge with a capacity of 50 L, at a velocity of 1380 rpm. This procedure, in addition to removing the lipids, also eliminated residues of casein and bacteria [16]. Then, cheese whey was pasteurised at a temperature of 65 °C for a period of 30 min.

The pretreated sheep cheese whey was concentrated by ultrafiltration until a volume concentration factor (VCF) of 3.0, using a plate and plane module M20, from Alfa Laval, Nakskov, Denmark. In all the experiments, the membranes used were ETNA10PP (purchased at Alfa Laval, Denmark), with a cutoff of 10 kDa and a total membrane surface area of 0.072 m^2^. The selection of membrane and operating conditions (transmembrane pressure of 2.0 × 10^5^ Pa and feed circulation velocity of 0.94 m·s^−1^) was performed through total recirculation experiments and is described elsewhere [17]. The temperature of filtration in all the experiments was kept at 25 °C.

#### 3.2.2. Samples of Goat Cheese Whey

**Figure 1:**
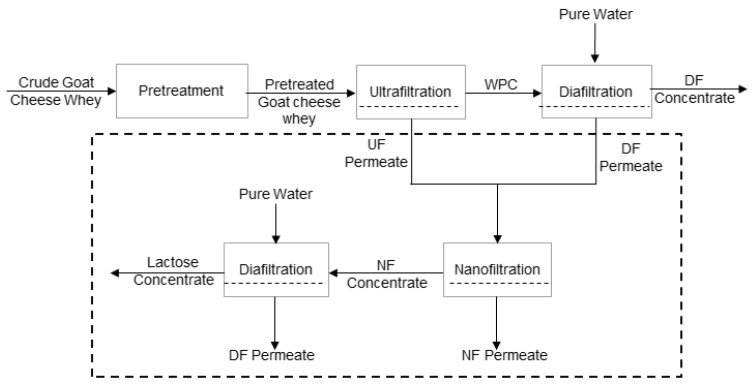
Schematic representation of the processes involved in this work.

The samples of goat cheese whey were subjected to a pretreatment identical to that used with sheep cheese whey up to the pasteurising stage. Then, goat cheese whey was ultrafiltrated until a VCF of 2.0, using the same module and total membrane surface area of 0.072 m^2^, but with membranes ETNA01PP, of the same material and lower cutoff (1 kDa). The selection of a narrower membrane was performed with the objective of enhancing nitrogen and lactose fraction separation, minimising losses of protein into the permeate stream, because goat cheese whey is poorer in whey proteins [6]. The same operating conditions of transmembrane pressure, feed circulation velocity, and temperature were used. After ultrafiltration, a discontinuous three-stage diafiltration process was carried out in the same operating conditions in order to remove lactose and minerals into the permeate stream. During the first and second stages, volumes of pure water (from reversis osmosis), corresponding to 20% of the volume of the retentate in the tank, were added; in the last stage, a pure water volume of 10% of the volume of the retentate in the tank was added. At each stage, the filtration process ran until the volume of the collected diafiltrate was equal to the volume of the initial water added. The membranes were not cleaned between each stage. This procedure was used in order to obtain a more purified nitrogenous fraction in the retentates and, on the other side, to allow a better recovery of a lactose demineralised fraction through the nanofiltration of ultrafiltration (UF) permeates. The permeates from ultrafiltration and dia-ultrafiltration (resulting from diafiltration of the ultrafiltration retentates) were mixed and homogenised, and then subjected to nanofiltration. The schematic representation of the processes involved is shown in Figure 1. In this figure, the designation of pure water refers to water obtained from reverse osmosis.

### 3.3. Physicochemical Characterisation of Samples

The samples (feed, retentates, and permeates) were analysed for: pH (by potentiometry); electrical conductivity (by condutimetry); relative density (with a lactodensimeter); viscosity at 25 °C (with a viscometer); lactose, according to the method described in Reference [18]; total solids by gravimetry [19]; total nitrogen, by the Kjeldahl reference method and crude protein, obtained from total nitrogen multiplied by the factor 6.38 [20] and adapted for cheese whey; nonprotein nitrogen, according to the Kjeldahl method, after precipitation of proteins by trichloroacetic acid at 12% [21]; total suspended solids, by gravimetry, according to the procedure described in Reference [22]; the fat content, determined by the Soxhlet extraction method, described in Reference [23]; sodium and potassium, by emission flame photometry, according to the procedure described by the author of [24]; calcium and magnesium by atomic absorption spectrophotometry with air–acetylene flame [25]; chloride, by volumetric precipitation, according to the method of Charpentier_Volhard [26]; phosphates, by the spectrophotometric method of ammonium molybdate [27]; chemical oxygen demand (COD), determined by the method described in Reference [28]; biochemical oxygen demand by the method described in Reference [29]; and total organic carbon (TOC) determinations, performed in a TOC analyser.

### 3.4. Nanofiltration Experiments

Nanofiltration experiments of the permeates of ultrafiltration, both from ultrafiltration of sheep and goat cheese whey, were performed using membranes NFT50(NF), from Alfa Laval, Navskov, Denmark, and with the same module used for ultrafiltration experiments. The total membrane surface area used was 0.072 m^2^. These membranes were commercialised by Alfa-Laval (Denmark) and are composite membranes with an active layer made of polyamide semiaromatic (polipiperazine), supported on two layers of polysulfone and polyester.

#### 3.4.1. Membrane Characterisation

Membranes were characterised in terms of water hydraulic permeability and cutoff. During the permeation of a pure solvent across a membrane, the volumetric permeate flux $J_{w}$ is given by the following equation:

(8) $$J_{w}=\left(L_{p}/\mu \right)\Delta P,$$

where $L_{p}$ (m) is the intrinsic permeability of the membrane, which only depends on its morphological characteristics; µ (Pa·s) is the viscosity of the solvent; and ΔP (Pa) is the applied transmembrane pressure. 

The determination of the hydraulic permeability of the nanofiltration membranes was performed by measuring the permeate fluxes of the pure water in a range of transmembrane pressures between 1.0 × 10^6^ Pa and 4.0 × 10^6^ Pa, while keeping the temperature (25 °C) and feed circulation velocity (0.94 m·s^−1^) constant. According to Equation (5), the proportionality constant is the hydraulic permeability or solvent permeability coefficient (m·s^−1^·Pa^−1^) and can be obtained experimentally from the slope of the calibration curve to an intercept equal to zero, represented by that equation.

The molecular weight cutoff (MWCO) is defined as the molar mass of a standard molecule whose rejection by the membrane is greater than 90.9% [15,30]. Its determination was carried out through permeation of dilute solutions (2.0 kg·m^−3^) of the reference solutes: Sodium chloride, magnesium sulphate, sodium sulphate, d-glucose, and lactose, at a transmembrane pressure of 9.0 × 10^5^ Pa and a feed circulation velocity of 0.94 m·s^−1^. During the experiments, samples of retentates and permeates were collected for analysis. In the case of the salts, the electrical conductivity of the solutions was measured and related with the concentration of the solutes. For the solutions of d-glucose and lactose, the concentrations of the solutes in the samples were determined by analysing the chemical oxygen demand (COD). The results obtained allowed the calculation of the apparent rejection coefficients, R:

R = 1 − C_p_/C_r_, (9)

where C_p_ is the concentration of COD in the permeate and C_r_ is the concentration of COD in the retentate, for each solute.

The MWCO was determined through the intersection of the straight line: Log (R/(1 − R)) = 1, corresponding to the molar mass of the solute that is rejected at 90.9%, with the straight line obtained by representing log (R/(1 − R)) as a function of the molar mass of the reference solutes, M.

#### 3.4.2. Nanofiltration Experiments in Total Recirculation Mode

The objectives of the experiments in total recirculation mode (the permeate and the retentate were both recirculated into the feed tank) were to evaluate the influence of the operating conditions, namely of transmembrane pressure and feed circulation velocity on the efficiency of the operation, measured in terms of permeate fluxes and of apparent rejection coefficients of some components, such as lactose and minerals.

These runs were performed with the ultrafiltration permeates of sheep cheese whey in the range of transmembrane pressure between 1.0 × 10^6^ and 3.0 × 10^6^ Pa, keeping the feed circulation velocity and temperature. Although a pressure of 4.0 × 10^6^ Pa was tested for concentrate in this sample (results not shown), the performance of the process was much lower due to the initial higher fluxes, leading to a much faster accumulation of solutes near the membrane surface, resulting in a very sharp decline of permeate fluxes. Two circulation velocities (0.94 m·s^−1^ and 1.42 m·s^−1^) were tested at 25 °C and the pH was measured during the experiments (6.06 ± 0.04). The stabilisation time at each transmembrane pressure was 30 min.

With respect to goat cheese whey, the feed used was a mixture of the ultrafiltration/diafiltration permeates (Figure 1) and the experiments were carried out in a range of transmembrane pressure between 5.0 × 10^5^ and 2.0 × 10^6^ Pa, keeping a feed circulation velocity of 0.94 m·s^−1^, a temperature of 25 °C, and a constant pH (5.43 ± 0.08) during all the runs. The total membrane area used in all the experiments was 0.072 m^2^.

#### 3.4.3. Nanofiltration Experiments in Concentration Mode

In nanofiltration experiments in concentration mode, only the retentate is recirculated, being the permeate collected into a beaker. In the assays performed with the UF permeates of sheep cheese whey, the effect of the concentration factor (up to a VCF of 2.5) on the permeate fluxes and apparent rejection coefficients was studied. The tests were carried out at a constant transmembrane pressure of 2.0 × 10^6^ Pa, a feed velocity of 1.42 m·s^−1^, a temperature of 25 °C, and pH of about 6.0. Samples of concentrates and permeates were collected for the following VCFs: 1.5; 1.75; 2.0; and 2.5.

The concentration experiments with the UF/DF permeates of goat cheese whey were performed until a VCF of 2.0, at a transmembrane pressure of 2.0 × 10^6^ Pa, at a feed recirculation velocity of 0.94 m·s^−1^, a temperature of 25 °C, and a pH of 5.43.

In all the NF experiments, the same kind of membranes (NFT50 or NF) and total surface area were used (0.072 m^2^). Samples of feed, retentates, and permeates were collected for analysis.

#### 3.4.4. Cleaning and Disinfection Cycle

Table 1 summarises the procedure used for the cleaning and disinfection of membranes following a long or short cycle, realised both before and after the experiments. The range of transmembrane pressure, feed circulation velocity, temperature, pH, and concentrations of the cleaning and disinfection chemicals followed the recommendations of the manufacturer for the specific membranes used. The new membranes were always subjected to the longest cycle, in such a way that their pure water hydraulic permeability reflected the most drastic cleaning and disinfection conditions needed after the experiments with the samples. In order to evaluate which cycle (longest or shortest) should be used after the experimental runs, membranes were washed three times with pure water and hydraulic permeability was measured and compared with that of the new membranes. If the initial water hydraulic permeability was recovered (at least until 95%), the shortest cycle was realised and, again, water hydraulic permeability was checked. The longest cycle was carried out in the cases where pure hydraulic permeability did not reach the intended recovery. Between each successive cleaning with chemicals, rinsing with distilled water was performed to remove the reagents used from the plant.

## 4. Results and Discussion

### 4.1. Physicochemical Characterisation of Sheep and Goat Cheese Whey

Table 2 presents the physicochemical characterisation of the following samples: Raw sheep cheese whey, pretreated sheep and goat cheese whey (before ultrafiltration), and permeates of ultrafiltration (feed for nanofiltration). The results shown are the average values obtained for the various parameters and their respective confidence intervals at 95%, calculated based on the Student’s *t* distribution, since the sample size is less than 30 [31].

The comparison of the composition of the pretreated sheep cheese whey (PSCW) with that of pretreated goat cheese whey (PGSW) allows the conclusion that sheep cheese whey has a higher total solids content, mainly due to its high concentration of crude protein. In fact, the concentration of whey proteins in sheep cheese whey is higher than that of goat or bovine cheese whey [6]. Although the concentration of fat in sheep cheese whey is also higher than that of goat cheese whey, it seems that the pretreatment used (filtration, skimming, and pasteurisation) was more efficient with sheep cheese whey than with goat cheese whey. This is because in PSCW, the fat concentration is 0.23 kg m^−3^, while in PGCW it is 3.90 kg m^−3^. Regarding the mineral composition of both pretreated cheese whey, it is noted that the dominant ions are sodium and chloride, in contrast to that of sweet bovine cheese whey, where potassium prevails [32,33]. These results are in agreement with the values observed by other authors [34,35] and can be justified by the addition of sodium chloride, both in milk and, later, during the manufacture of cheese. 

When comparing the composition of permeate of ultrafiltration of PSCW (PUF-S) and permeate of ultrafiltration of PGCW (PUF-G), we can see major differences between them. The permeates resulting from the ultrafiltration of sheep cheese whey contain higher concentrations of nitrogen, and therefore crude protein, lactose, calcium, magnesium, phosphorus, and potassium. The permeates of the ultrafiltration of goat cheese whey have higher concentrations of fat and chloride. These results are probably due to the following reasons: (i) Different compositions of the initial samples (feeds subjected to ultrafiltration); (ii) the membranes used in production of PUF-S have a cutoff of 10 kDa (larger pores), while in PUF-G the cut-off of the membranes is of 1 kDa; the sample PUF-G is diluted because IT was obtained by mixing the permeates of UF with the permeates from UF/DF, as shown in Figure 1.

### 4.2. Characterisation of Nanofiltration Membranes

**Figure 2:**
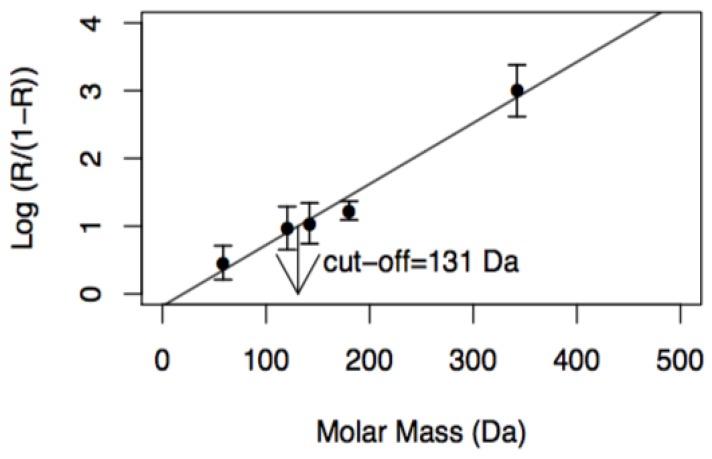
Experimental determination of molecular weight cutoff (MWCO) of NF membranes at a transmembrane pressure of 1.0 × 10^6^ Pa, v = 0.94 m·s^−1^ and concentrations of reference solutes of 2.0 kg m^−3^.

NF membranes were characterised in terms of pure water hydraulic permeability and molecular weight cutoff. The results are shown in Figure 2 and Table 3.

Figure 2 shows the graphical determination of the MWCO of NF membranes in accordance with the method described in Section 3.4.1. The cutoff obtained, 131 Da, is close to that determined by other authors (150 Da) [36].

Table 3 presents the linear regression lines (with 95% confidence intervals and correlation coefficients) used for the determination of pure water permeability and the MWCO of NF membranes. The hydraulic permeability is also close to that obtained from other researchers [37,38] for membranes with the same reference and with similar operating conditions.

### 4.3. Total Recirculation Mode Experiments

#### 4.3.1. Influence of Transmembrane Pressure and Feed Circulation Velocity on Permeate Fluxes

**Figure 3:**
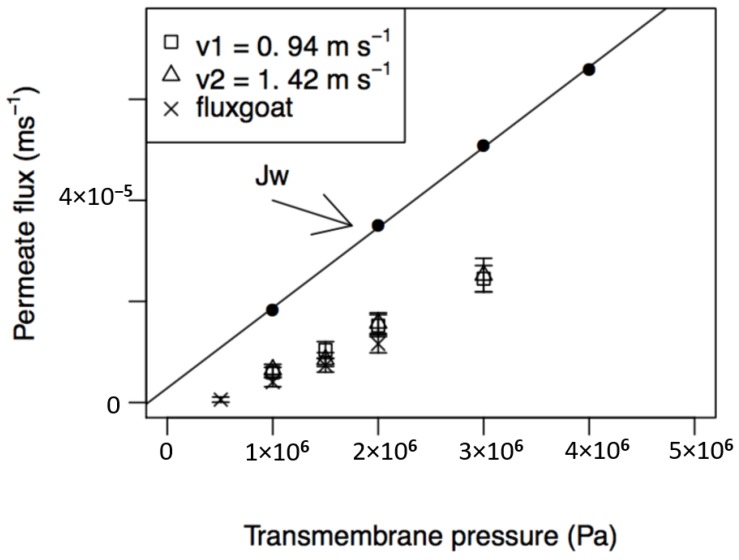
Influence of transmembrane pressure and feed circulation velocities on permeate fluxes, at a temperature of 25 °C, for PUF-S (at v_1_ = 0.94 m·s^−1^ and v_2_ = 1.42 m·s^−1^) and PUF-G (at v_1_ = 0.94 m·s^−1^).

The influence of transmembrane pressure and feed circulation velocity on permeate fluxes from the nanofiltration of PUF-S and PUF-G and their comparison with pure water fluxes is presented in Figure 3. The permeate fluxes, for both samples, increase linearly with the transmembrane pressure in all ranges of pressure analysed and are lower for PUF-G. The equations of the regression lines obtained with the permeates from PUF-S (J_sheep_) and from PUF-G (J_goat_), with the indication of 95% confidence intervals for the estimated parameters (slope and intercept), the sample size (n), the correlation coefficient (ρ), and level of significance (p), are:

J_sheep_ = (9.28 × 10^−10^ ± 2.24 × 10^−13^) ΔP + (−3.40 × 10^−6^ ± 4.49 × 10^−7^) (12)

$$\left(n\;=\;12;\;\rho =0.999;\;p=5.15\times 10^{-13}\right)$$

J_goat_ = (6.98 × 10^−12^ ± 4.23 × 10^−13^) ΔP + (−3.30 × 10^−6^ ± 3.21 × 10^−7^) (13)

$$\left(n\;=\;8;\;\rho =0.998;\;p=6.15\times 10^{-13}\right)$$

With respect to the influence of feed circulation velocity on permeate fluxes of PUF-S, it can be observed that they are very close for both velocities studied (0.94 and 1.42 m·s^−1^). This result may indicate that mass transfer near the membrane surface does not control the process.

The permeate fluxes obtained, both with PUF-S and PUF-G, are always lower than the corresponding water fluxes in all the pressure ranges studied. The main reasons for this behaviour can be attributed to the following phenomena: High osmotic pressures of the permeates, concentration polarisation, and fouling phenomena. The effect of high osmotic pressures leads to a decrease in the effective transmembrane pressure and, therefore, to lower permeate fluxes. This factor is likely to be the most important when dealing with the nanofiltration of ultrafiltration permeates of cheese whey due to the high concentrations of lactose and salts, such as chloride, sodium, and potassium, which are the major constituents of these permeates (Table 2). This is in accordance with that described elsewhere [13]. Using the regression lines (Equations (12) and (13)) and extrapolating to zero permeate flux, we can estimate an osmotic pressure of 3.66 × 10^5^ Pa for PUF-S and 4.73 × 10^5^ Pa for PUF-G. However, this value is likely underestimated, because typical osmotic pressures measured in cheese whey are around 7.0 × 10^5^ Pa [14] and some researchers [38] obtained experimentally osmotic pressures of 6.1 × 10^5^ Pa at 10 °C. The evaluation of the relative importance of these phenomena is presented in Section 4.5.

#### 4.3.2. Influence of Transmembrane Pressure and Feed Circulation Velocity on Apparent Rejection Coefficients

The influence of transmembrane pressure and feed circulation velocity on apparent rejection coefficients is shown in Table 4. During the nanofiltration of PUF-S, membranes were almost totally retentive with respect to lactose.

When analysing the influence of transmembrane pressure on apparent rejection coefficients of total solids and COD, it is observed that they are independent of pressure and feed circulation velocity, having an average of 0.88 for total solids and 0.99 for COD. This high rejection to organic matter is likely due to the high rejection of lactose by membranes, since this component is the main contributor of organic load in these samples.

With respect to nitrogen, it can be observed that apparent rejection coefficients are in the range between about 0.5 and 0.7, which means that part of the nitrogen is not in the form of protein, but as low molecular nonprotein nitrogen compounds, such as small peptides and amino acids. Apparent rejections increase with applied transmembrane pressure, being slightly lower at the higher feed circulation velocity (1.42 m·s^−1^). In relation to salts, the apparent rejection coefficients increase proportionally with transmembrane pressure, from about 0.3 until 0.6, and are close for the two circulation velocities. These results indicate that the increase in pressure may have contributed to the growth of a polarisation layer and, therefore, to the increase of apparent rejection coefficients. The transmembrane pressure seems to be the most important operating variable for the control of the rejections of both the nitrogen compounds and salts.

Relative to apparent rejection coefficients obtained during the nanofiltration of PUF-G, it was observed in the range of transmembrane pressure used (between 5.0 × 10^5^ Pa and 2.0 × 10^6^ Pa) that apparent rejections of total solids, lactose, and total nitrogen increase with pressure in the following intervals: 0.5–0.7, 0.7–0.95, and 0.4–0.7, respectively. In this case, the apparent rejections of lactose are lower than those observed during the experiments with PUF-S (Table 4) and increase with transmembrane pressure. The lower content of total solids and also lactose (Table 2) probably facilitated the removal of lactose into the permeate stream.

### 4.4. Concentration Mode Experiments

#### 4.4.1. Nanofiltration of Permeates from Sheep Cheese Whey

Based on the results discussed in Section 4.3, the concentration experiments with PUF-S were carried out at a transmembrane pressure of 3.00 × 10^6^ Pa, because the permeate fluxes are higher at this pressure. The feed circulation velocity used in concentration experiments was 1.42 m·s^−1^. Although permeate fluxes at the two analysed velocities are very close (shown in Figure 3), the apparent rejection coefficients of smaller compounds are slightly lower at the higher velocity and, therefore, the use of this velocity (higher turbulence near the membrane surface) can promote a decrease in polarisation layer, enhancing the performance of the separation process.

**Figure 4:**
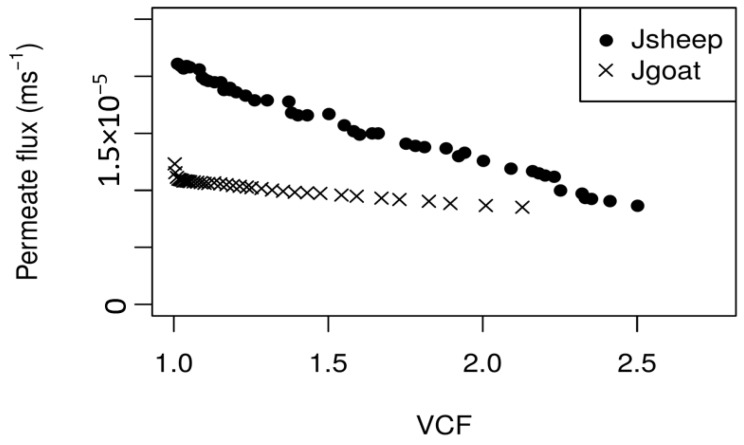
Variation of average (3 replicates) permeate fluxes with the volume concentration factor (VCF) for the concentration by nanofiltration of PUF-S (ΔP = 3.0 × 10^6^ Pa; <v> = 1.42 m·s^−1^) and PUF-G (ΔP = 2.0 × 10^6^ Pa; <v> = 0.94 m·s^−1^), at T = 25 °C.

Figure 4 shows the evolution of permeate flux (average values) during the NF concentration process, for both PUF-S and PUF-G. It can be observed that with PUF-S, permeate fluxes decrease sharply with VCF during the concentration process, which is completed when a VCF = 2.5 is reached (after 2 h 15 min). The permeate flux decline over the total experiment time was approximately 60%. The reduction of the permeate fluxes with VCF can be attributed to the following factors: Increased osmotic pressure, mainly due to the increased concentration of lactose and salts; and concentration polarisation and/or fouling of the membranes, due to the formation of insoluble salts, such as calcium phosphates [13]. The use of a higher transmembrane pressure (3.00 × 10^6^ Pa) for the nanofiltration of PUF-S allowed a higher permeate flux at the very beginning of the experiments. However, it also led to a greater accumulation of lactose and salts near the membrane surface more rapidly, thus promoting a higher osmotic pressure (reducing the effective pressure), and increased concentration polarisation phenomena, mainly due to high retention lactose. This effect is still enhanced because lactose concentration in PUF-S is higher than that of PUF-G, as is its retention by NF membranes. Furthermore, as both pH (6.06) of the PUF-S samples and concentrations of both calcium and phosphate are higher, it is also possible that the formation of insoluble salts of calcium phosphates contributed to a further decline on permeate fluxes [32].

During the concentration process of PUF-S and until the analysed VCF (2.5), the apparent rejection coefficients of total solids, total nitrogen (and consequently crude protein), nonprotein nitrogen, and lactose were almost constant. The average apparent rejection coefficients for total solids, lactose, total nitrogen, and nonprotein nitrogen are: 0.920, 0.995, 0.768, and 0.738, respectively. These high rejections of organic matter by NF membranes led to a final permeate with a much lower organic load.

With respect to the apparent rejections of the ions, it was observed that the rejections of bivalent ions (calcium, magnesium, and phosphates) are higher than 95%, likely because they are determined by the steric hindrance of the ion at the entrance of the pore. In addition, since the NF membranes used have an isoelectric pH of 4.2 [36] and the pH of our feed (PUF-S) is 6.06, the membranes present a negative charge, which may have favoured the formation of complexes between bivalent ions through electrostatic interactions [39].

The apparent rejections of the monovalent ions, sodium, potassium, and chloride were much lower, being about 48%, 18%, and 20%, respectively. Therefore, the final permeate is enriched in these ions and its use during the cheese making process (e.g., to replace the brine usually added to the curd or during the ripening process of the cheese) will be further investigated.

The final NF retentates are enriched in lactose and nitrogen compounds, but still have a high concentration of minerals, namely calcium and magnesium. Thus, its use for food or pharmaceutical industry and/or to produce energy are possible uses of these kind of samples, after a previous evaluation based on technical and economic reasons.

#### 4.4.2. Nanofiltration of Permeates from Goat Cheese Whey

The concentration of PUF-G by nanofiltration was carried out at the highest transmembrane pressure tested, 2.0 × 10^6^ Pa, because permeate fluxes were higher at this pressure, as they were apparent rejection coefficients of lactose. The feed circulation velocity used was 0.97 m·s^−1^.

Figure 4 shows a great difference between the evolutions of permeate fluxes with VCF for PUF-S and PUF-G. Thus, at the beginning of the experiments, the permeate fluxes from the nanofiltration of PUF-G are much lower (around two times) than those of PUF-S. However, after the first few minutes (around 3 min), the decrease was much smoother until the end of the experiment (only 22% in contrast to 60%, obtained during the NF of PUF-S), when VCF = 2.13 was reached, corresponding to a time of approximately 2.5 h. At VCF = 2.13, both permeate fluxes are much closer. The better stabilisation of the permeate fluxes from PUF-G can be attributed to the lower concentration of lactose and salts (except for the concentration of chloride ions), partially due to the effect of dilution caused by the process of diafiltration, thus leading to a less pronounced increase in osmotic pressure. Furthermore, since the pH of these samples is lower than that of PUF-S (5.43 in contrast to 6.06 of PUF-S), the possibility of fouling caused by the accumulation of insoluble salts, namely of calcium phosphates on the membrane, is less probable.

In spite of its higher stability, permeate fluxes obtained with PUF-G are lower than those obtained with PUF-S until the VCF studied. This is likely due to the following reasons: The lower transmembrane pressure used and the higher initial concentration of chloride ions in PUF-G (8.70 kg·m^−3^), comparatively to that of PUF-S (5.20 kg·m^−3^), giving rise to a greater osmotic pressure (according with van’t Hoff equation) at the very beginning of the experiments and a greater reduction of effective pressure.

The apparent rejection coefficients of lactose, total organic carbon (TOC), and crude protein increased during the process of NF–DF in the following range: From 94% to 96%, for lactose; from 92% to 94%, for TOC; and from 65% to 75%, for crude protein. By contrast, the rejections of nonprotein nitrogen compounds (NPN) decreased during the process of NF–DF from 75% to 67%, which indicates that the diafiltration process partially removed lower molecular nonprotein nitrogen compounds, probably amino acids.

With respect to mineral composition, as expected, it was observed that the apparent rejections of the bivalent ions (calcium, magnesium, and phosphate) were high and increased during the diafiltration process (more than 80%). However, the apparent rejection coefficients of the monovalent ions (sodium, potassium, and chloride) were low (about 20%) and the diafiltration process allowed their removal for the final permeates.

These results indicate that most of the organic matter, mainly present as lactose and nitrogen compounds, is removed by the NF–DF process in the retentates, thus reducing the environmental impact of cheese whey.

### 4.5. Application of a Mass Transfer Model to the Permeates of Nanofiltration of PUF-S

The application of a simple linear model to peer J_exp_ and C_lacb_ values yields the following equation for the regression line (n = 46; ρ = 0.994; p < 2.2 × 10^−16^):

J_sheep_ = (−6.50 × 10^−8^ ± 2.18 × 10^−9^) C_lacb_ + (2.88 × 10^−5^ ± 4.50 × 10^−7^). (14)

Comparing this equation with that of the model described by Equation (7) (Section 3), the total mean resistance (R_t_) can be determined through the ordinate at the origin and the mean concentration polarisation module, β, from the respective slope of the line. The value of K, which represents the difference in osmotic pressure across the membranes due to chloride ions, was calculated from experimental values and is 8.26 × 10^4^ Pa. Knowing the intrinsic membrane resistance (6.64 × 10^13^ m^−1^), as shown in Table 3, it is possible to calculate the sum of the average resistances to concentration polarisation (R_cp_) and to fouling (R_f_). The results obtained are summarised in Table 5.

Comparing the values of all the resistances to transport across the membranes, it can be observed that the membrane presents the major resistance. However, the polarisation concentration modulus reveals that, on average, the concentration of lactose in the polarisation layer close to the membrane surface is more than twice that in the bulk solution. Therefore, the effect of the concentration of lactose in the polarisation layer contributes significantly to the osmotic pressure and this effect seems to be determinant in the decline of permeate fluxes.

## 5. Conclusions

The performance of the nanofiltration of permeates of ultrafiltration from sheep cheese whey is mainly affected by the higher concentrations of lactose and salts (especially sodium chloride). However, since lactose is almost totally retained, in contrast to chloride ions that preferentially permeate the membranes, the increase of the concentration of lactose during the concentration process has a major impact on the decline of the permeate fluxes. This leads to an increase in osmotic pressure and, consequently, a reduced effective pressure. The model of mass transfer reasonably describes the experimental behaviour observed, thus confirming this conclusion.

The nanofiltration of the permeates of goat cheese whey is a more stable process, but the permeate fluxes are lower until the VCF studied. Both the use of a lower transmembrane pressure (2.0 × 10^6^ Pa) and the effect of dilution, caused by diafiltration, may have contributed to the lower concentrations of lactose and salts near the membrane surface, thus leading to a smaller increase in osmotic pressure during the runs and, consequently, to a much smoother decrease of permeate fluxes.

Based on the results obtained, some procedures can be tested in our future work in order to enhance the performance of the nanofiltration process of PUF-S. The application of a lower transmembrane pressure, such as 2.0 × 10^6^ Pa, and/or the use of nanodiafiltration, can avoid a very fast and great increase of the lactose and salts concentration near the membrane surface, allowing better control of the osmotic pressure increase and the decline of the permeate fluxes. In addition, the dilution caused by the previous diafiltration process also decreases the concentration of calcium and phosphate ions, minimising the possible effects of mineral fouling.

## Figures and Tables

**Table 1:** Process of cleaning and disinfection of nanofiltration membranes ^a^.

Experimental Conditions	Short Cycle	Long Cycle	Time (min)
Operational parameters			
Transmembrane pressure (Pa)	1 × 10^6^	1 × 10^6^	-
Feed circulation velocity (m·s^−1^)	0.92	0.92	-
Temperature (°C)	25	25	-
Cleaning			
pH	2–11	2–11	-
NaOH (w/v %)	-	0.05	15
Na-EDTA (w/v %)	-	0.20	15
HNO_3_ (w/v %)	-	0.10	15
C_6_H_8_O_7_ (w/v %)	-	0.50	15
Disinfection			
H_2_O_2_ (mg·L^−1^) at 25 °C	1000	1000	30

^a^ Since pH limits dominate, adjustments were made to the right value; Na-EDTA, ethylenediaminetetra-acetic acid, sodium salt.

**Table 2:** Physicochemical characterisation of sheep and goat cheese whey ^1^ (average values ± 95% confidence interval).

Parameter	Samples				
	RSCW	PSCW	PUF-S	PGCW	PUF-G
pH (25 °C)	5.62 ± 0.29	5.58 ± 0.28	6.06 ± 0.04	5.42 ± 0.22	5.43 ±0.08
K^25 °C^ (S·m^−1^)	2.09 ± 0.07	2.10 ± 0.07	-	2.51 ± 0.23	2.56 ± 0.06
ST (kg·m^−3^)	108.34 ± 4.74	87.34 ± 2.36	52.93 ± 0.64	77.94 ± 0.25	38.36 ± 0.01
NKjeldahl (kg·m^−3^)	2.777 ± 0.121	2.682 ± 0.109	0.605 ± 0.059	1.222 ± 0.120	0.251 ± 0.030
Crude protein (kg·m^−3^)	17.74 ± 0.77	17.10 ± 0.70	3.86 ± 0.38	7.80 ± 0.13	1.60 ± 0.30
NPN (kg·m^−3^)	-	-	0.490 ± 0.034	1.100 ± 0.020	0.200 ± 0.010
Lactose (kg·m^−3^)	52.0 ± 0.9	52.1 ± 1.0	41.6 ± 1.1	49.9 ± 0.60	38.9 ± 0.5
Fat (kg·m^−3^)	20.79 ± 4.12	0.23 ± 0.04	0.08 ± 0.01	3.90 ± 0.60	3.30 ± 0.02
Na (kg·m^−3^)	7.138 ± 0.260	7.142 ± 0.260	3.389 ± 0.141	4.555 ± 0.629	3.468 ± 0.350
K (kg·m^−3^)	0.993 ± 0.045	0.991 ± 0.042	1.093 ± 0.100	1.145 ± 0.045	0.849 ± 0.118
Ca (kg·m^−3^)	0.492 ± 0.023	0.474 ± 0.004	0.396 ± 0.019	0.289 ± 0.028	0.178 ± 0.002
Mg (kg·m^−3^)	0.089 ± 0.005	0.087 ± 0.005	0.102 ± 0.005	0.076 ± 0.002	0.054 ± 0.001
Cl (kg·m^−3^)	7.44 ± 0.44	7.54 ± 0.47	5.20 ± 0.070	9.82 ± 0.58	8.70 ± 0.467
Phosphate (kg·m^−3^)	1.43 ± 0.16	1.46 ± 0.15	0.43 ± 0.08	0.35 ± 0.12	0.046 ± 0.012

^1^ RSCW = raw sheep cheese whey; PSCW = pretreated sheep cheese whey (before ultrafiltration); PUF-S = permeate of ultrafiltration of PSCW (feed for nanofiltration); PGCW = pretreated goat cheese whey (before ultrafiltration); PUF-G = permeate of ultrafiltration of PGCW (feed for nanofiltration).

**Table 3:** Determination of hydraulic permeability and cutoff of nanofiltration (NF) membranes.

Regression Lines (with 95% CI for the Parameters Estimated)	Hydraulic Permeability (ms^−1^Pa^−1^)	Intrinsic Hydraulic Permeability, Lp (m)
J_w_ = ($1.68\times 10^{-11}\pm 4.98\times 10^{-13})$·ΔP (10) R = 0.999	1.68 × 10^−11^	1.51 × 10^−14^
Log (R/(1 − R)) = ($9.00\times 10^{-3}$± $4.98\times 10^{-5}$) + (−$1.77\times 10^{-1}$ ± $2.01\times 10^{-3}$)·M (11) R = 0.990	-	-

**Table 4:** Apparent rejection coefficients to nitrogen N (kjeldahl), salts, and chemical oxygen demand (COD) obtained during nanofiltration of PUF-S, at different transmembrane pressures, feed circulation velocities, and a temperature of 25 °C.

ΔP (Pa)	<v> (m·s^−1^)	R_N_	R_salts_	R_COD_
1.00 × 10^6^	0.94	0.576 ± 0.015	0.260 ± 0.015	0.992 ± 0.001
1.00 × 10^6^	1.42	0.456 ± 0.012	0.249 ± 0.007	0.994 ± 0.001
1.50 × 10^6^	0.94	0.612 ± 0.012	0.377 ± 0.010	0.994 ± 0.001
1.50 × 10^6^	1.42	0.553 ± 0.013	0.362 ± 0.015	0.996 ± 0.001
2.00 × 10^6^	0.94	0.632 ± 0.010	0.480 ± 0.004	0.995 ± 0.000
2.00 × 10^6^	1.42	0.568 ± 0.013	0.437 ± 0.015	0.995 ± 0.001
3.00 × 10^6^	0.94	0.666 ± 0.008	0.551 ± 0.003	0.996 ± 0.001
3.00 × 10^6^	1.42	0.587 ± 0.005	0.528 ± 0.013	0.995 ± 0.001

**Table 5:** Estimates of average total resistance (±RSD ^1^), resistance due to concentration polarisation, fouling resistance, and concentration polarisation modulus (±0.09 RSD), obtained by applying the model described by the Equation (7).

R_t_ (m^−1^)	(R_cp_ + R_f_) (m^−1^)	β
1.08 × 10^14^ ± 5.93 × 10^−2^	4.16 × 10^13^	2.35 ± 0.09

^1^ RSD = relative standard deviation.
